# Leisure‐time physical activity and sarcopenia among older adults from low‐ and middle‐income countries

**DOI:** 10.1002/jcsm.13215

**Published:** 2023-03-05

**Authors:** Louis Jacob, Razak M. Gyasi, Hans Oh, Lee Smith, Karel Kostev, Guillermo F. López Sánchez, Masoud Rahmati, Josep Maria Haro, Mark A. Tully, Jae Il Shin, Dong Keon Yon, Ai Koyanagi

**Affiliations:** ^1^ Research and Development Unit Parc Sanitari Sant Joan de Déu, CIBERSAM, ISCIII Barcelona Spain; ^2^ Department of Physical Medicine and Rehabilitation Lariboisière‐Fernand Widal Hospital, AP‐HP, University Paris Cité Paris France; ^3^ African Population and Health Research Center Nairobi Kenya; ^4^ National Centre for Naturopathic Medicine, Faculty of Health Southern Cross University Lismore New South Wales Australia; ^5^ Suzanne Dworak Peck School of Social Work University of Southern California Los Angeles CA USA; ^6^ Centre for Health, Performance, and Wellbeing Anglia Ruskin University Cambridge UK; ^7^ University of Marburg Marburg Germany; ^8^ Division of Preventive Medicine and Public Health, Department of Public Health Sciences, School of Medicine University of Murcia Murcia Spain; ^9^ Department of Physical Education and Sport Sciences, Faculty of Literature and Human Sciences Lorestan University Khorramabad Iran; ^10^ School of Medicine Ulster University Coleraine UK; ^11^ Department of Pediatrics Yonsei University College of Medicine Seoul Republic of Korea; ^12^ Center for Digital Health, Medical Science Research Institute Kyung Hee University College of Medicine Seoul Republic of Korea; ^13^ Institució Catalana de Recerca i Estudis Avançats (ICREA) Barcelona Spain

**Keywords:** epidemiology, leisure‐time physical activity, low‐ and middle‐income countries, older adults, sarcopenia

## Abstract

**Background:**

There are no data on the association between leisure‐time physical activity (LTPA) and sarcopenia in older adults from low‐ and middle‐income countries (LMICs). This study aimed to investigate the association between LTPA and sarcopenia in individuals aged ≥65 years living in six LMICs.

**Methods:**

Cross‐sectional data from the Study on Global AGEing and Adult Health (China, Ghana, India, Mexico, Russia and South Africa) were analysed. Sarcopenia referred to the presence of both low skeletal muscle mass and weak handgrip strength. LTPA was assessed using the Global Physical Activity Questionnaire and was analysed as a dichotomized variable [high LTPA (>150 min/week of moderate‐to‐vigorous LTPA) or low LTPA (≤150 min/week)]. Multivariable logistic regression analysis was conducted to assess associations.

**Results:**

There were 14 585 individuals included in this study [mean (SD) age 72.6 (11.5) years; 55.0% women]. The prevalence of high LTPA and sarcopenia was 8.9% and 12.0%, respectively. After adjusting for potential confounders, low LTPA was significantly associated with higher odds for sarcopenia [prevalence odds ratio (POR) = 1.85, 95% confidence interval (CI) = 1.29–2.65] compared with high LTPA. Significant associations were found in women (POR = 3.22, 95% CI = 1.82–5.68) but not in men (POR = 1.52, 95% CI = 0.99–2.35).

**Conclusions:**

A positive and significant association between low LTPA and sarcopenia was found among older adults from LMICs. The promotion of LTPA among older adults in LMICs may aid in the prevention of sarcopenia, especially among females, pending future longitudinal research.

## Introduction

Sarcopenia is an age‐related condition characterized by the progressive loss of skeletal muscle mass (SMM) and function, and this loss is one of the most critical changes related to ageing.^1^ Sarcopenia is now recognized as a muscle disease and is included in the International Classification of Diseases. The prevalence of sarcopenia is high in older adults and can reach 25% in those living in low‐ and middle‐income countries (LMICs).^2^ Given that the worldwide population is ageing rapidly, where the number of people ≥ 60 years is projected to increase from current figures of around 1 billion to 1.4 billion by 2030 and 2.1 billion by 2050,^3^ the prevalence of sarcopenia is expected to increase substantially in the coming decades. This is a major problem as sarcopenia is associated with higher risks of physical disability,^4^ impaired mental health^5^ and premature mortality.^6^ In this context, it is of utmost importance to identify the risk factors for sarcopenia, particularly in LMICs where the treatment and management of sarcopenia may be limited.

In recent decades, literature has shown that physical activity may protect against sarcopenia. Physical activity can be defined as body movements produced by skeletal muscles with the expenditure of energy.^7^ Physical activity may prevent sarcopenia via an increase in muscle mass and strength, prevention of several chronic physical conditions (e.g., cardiovascular diseases, dementia and diabetes)^8^ and polypharmacy,^9^, ^10^ as well as decreased risk of hospitalization.^11^, ^12^ Indeed, a 2017 systematic review and meta‐analysis of 25 studies revealed that physical activity was associated with a significant decrease in the odds for sarcopenia [odds ratio (OR) = 0.45, 95% confidence interval (CI) = 0.37–0.55].^13^ However, currently, there is very limited information on the effects of specific domains of physical activity on sarcopenia. There are several domains of physical activity, such as leisure‐time, occupational and travel‐related physical activity. It is possible for leisure‐time physical activity (LTPA) to be particularly important for the prevention of sarcopenia. Indeed, LTPA may be the most effective way to increase overall physical activity, especially in older age, as many older people are less likely to engage in occupational physical activity or active travel (e.g., walking and cycling). Moreover, levels of LTPA in older adults living in LMICs may be particularly low, as there frequently is a lack of sports facilities and insufficient information delivered to the general population on the benefits of regular LTPA in these countries, highlighting the importance of focusing on this type of physical activity in older people from these settings.^14^ To the best of the authors' knowledge, only two studies have focused on the effects of LTPA on sarcopenia, and in both studies, data were collected in high‐income countries (i.e., Italy^15^ and Spain^16^). These bodies of research identified an inverse and significant association between LTPA and sarcopenia. Given that low levels of LTPA are common in LMICs^17^ and that there is a significant relationship between LTPA and sarcopenia reported from high‐income countries, interventions to increase LTPA among people in LMICs may have a large potential for the prevention of sarcopenia. However, data are lacking from LMICs, and thus, more data on this topic from this setting are needed.

Therefore, this study aimed to investigate the association between LTPA and sarcopenia in older adults living in six LMICs. Given that most older people reside in LMICs,^3^ identifying risk factors for sarcopenia in these regions of the world is a public health priority.

## Methods

### The survey

This study used data from the Study on Global AGEing and Adult Health (SAGE), a survey undertaken in China, Ghana, India, Mexico, Russia and South Africa between 2007 and 2010. These countries were all classified as LMICs based on the World Bank classification at the time of the survey. The methodology of the survey has been described extensively in the literature.^18^ Briefly, nationally representative samples were obtained using a multistage clustered sampling design method. Samples included individuals aged ≥18 years, and adults aged ≥50 years were oversampled. Face‐to‐face interviews were conducted by trained staff with the use of a standard questionnaire. Questionnaires were translated based on a standard procedure to allow comparability between the participating countries. The survey response rates ranged from 53% for Mexico to 93% for China. Population structure, as reported by the United Nations Statistical Division, was further used to construct sampling weights. Finally, the World Health Organization (WHO) Ethical Review Committee and local ethics research review boards provided ethical approval, and each participant gave written informed consent.

### Sarcopenia

Based on the criteria of the revised European consensus on the definition and diagnosis of sarcopenia,^19^ sarcopenia corresponded to the presence of both low SMM and weak handgrip strength. SMM was calculated using the equation developed by Lee et al.: SMM = 0.244 * weight + 7.8 * height + 6.6 * sex − 0.098 * age + race − 3.3 [where sex = 0 (female) and sex = 1 (male); race = 0 (White and Hispanic), race = 1.4 (Black) and race = −1.2 (Asian)].^20^ Skeletal mass index (SMI) was further obtained by dividing SMM by body mass index (BMI) based on measured weight and height.^21^ Low SMM corresponded to the lowest quintile of sex‐stratified SMI values.^22^ Given that there may be racial differences in body composition,^23^ low SMI was determined using country‐specific cut‐offs. Finally, the average of two handgrip measurements of the dominant hand was used to define handgrip strength, and weak handgrip strength corresponded to <27 kg in males and <16 kg in females.^19^ As this cut‐off for handgrip strength was based on European studies, we also conducted a sensitivity analysis where weak handgrip strength was defined as the lowest tertile of handgrip strength based on sex‐ and country‐stratified values.

### Leisure‐time physical activity

The assessment of LTPA relied on questions from the Global Physical Activity Questionnaire (GPAQ) on sports, fitness, and leisure and recreational activities.^24^ Participants were asked how many days they do vigorous‐intensity sports, fitness or recreational (leisure) activities in a typical week and how much time they spend doing such activities on a typical day (e.g., running or playing football). Similar questions were asked for moderate‐intensity sports, fitness or recreational (leisure) activities (e.g., brisk walking, cycling or swimming). Time spent in moderate‐to‐vigorous LTPA per week was calculated based on these questions, and this time was dichotomized as high LTPA (i.e., >150 min/week) or low LTPA (i.e., ≤150 min/week) as in a previous SAGE publication.^25^ In some analyses, moderate‐to‐vigorous LTPA was used as a continuous variable (h/week).

### Control variables

Control variables were selected based on past literature^13^ and included age (years), sex (female or male), country‐wise wealth quintiles based on income, highest level of education achieved (≤primary, secondary or tertiary), BMI, number of chronic conditions, activities of daily living (ADL) difficulty, smoking (never, past or current), alcohol consumption in the past 30 days (yes or no), occupational physical activity and active travel. BMI corresponded to measured weight in kilograms divided by measured height in metres squared and was used as a four‐category variable [<18.5 (i.e., underweight), 18.5–24.9 (i.e., normal weight), 25.0–29.9 (i.e., overweight) and ≥30 kg/m^2^ (i.e., obesity)]. Eleven chronic physical diseases were documented: angina, arthritis, asthma, chronic back pain, chronic lung disease, diabetes, edentulism, hearing problem, hypertension, stroke and visual impairment. *Table* *S1* provides details on the diagnosis of these 11 conditions. The number of chronic conditions was included in the analyses as a three‐category variable (0, 1 or ≥2 chronic conditions). The assessment of ADL difficulty relied on questions about six standard basic ADL.^26^, ^27^, ^28^ All questions began with the introductory phrase ‘overall in the last 30 days, how much difficulty did you have’ and continued with the following: ‘in washing your whole body?’; ‘in getting dressed?’; ‘with moving around inside your home?’; ‘with eating (including cutting up your food)?’; ‘with getting up from lying down?’; and ‘with getting to and using the toilet?’. The answers were ‘none’, ‘mild’, ‘moderate’, ‘severe’ and ‘extreme/cannot do’. ADL difficulty was used as a dichotomous variable, and people answering ‘severe’ or ‘extreme/cannot do’ to at least one of the six questions were considered to have ADL difficulty.^29^ Finally, the GPAQ was used to calculate the amount of domain‐specific physical activity,^24^ and occupational physical activity and active travel were included as dichotomous variables (>150 or ≤150 min/week) as in previous SAGE studies.^25^, ^30^

### Statistical analysis

The statistical analysis was undertaken using Stata 14.2 (StataCorp LP, College Station, TX). Given that sarcopenia is an age‐related condition, the analysis only included adults aged ≥65 years. Differences in the characteristics of the sample by min/week of LTPA (>150 vs. ≤150 min/week) were tested by chi‐squared tests for categorical variables and Student's *t* tests for continuous variables. The association between LTPA (exposure) and sarcopenia (outcome) was studied using a multivariable logistic regression model. LTPA was included in the model as a dichotomous variable (i.e., >150 or ≤150 min/week) or as a continuous variable (h/week). We also conducted analysis using the two components of sarcopenia as separate outcomes (i.e., low SMM and weak handgrip strength). The analysis was done using the overall sample and sex‐stratified samples. All regression analyses were adjusted for age, sex, wealth, education, BMI, number of chronic conditions, ADL difficulty, smoking, alcohol consumption, occupational physical activity, active travel and country, except for the sex‐stratified analysis, which was not adjusted for sex. Adjustment for country was done by including dummy variables for each country in the model as in previous SAGE publications.^31^, ^32^ Predicted probability of sarcopenia by h/week of LTPA was also calculated based on a model adjusted for age, sex, wealth, education, BMI, number of chronic conditions, ADL difficulty, smoking, alcohol consumption, occupational physical activity, active travel and country, using mean values. The sample weighting and the complex study design were considered in all analyses. Results from the regression analyses are displayed as prevalence OR (POR) and 95% CI. The level of statistical significance was set at *P* value < 0.050.

## Results

**Figure 1:**
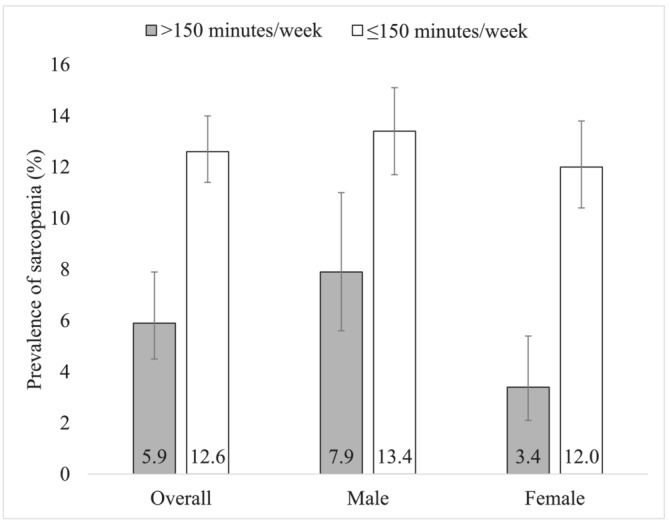
Prevalence of sarcopenia by min/week of leisure‐time physical activity (overall and by sex). Bars denote 95% confidence interval.

**Figure 2:**
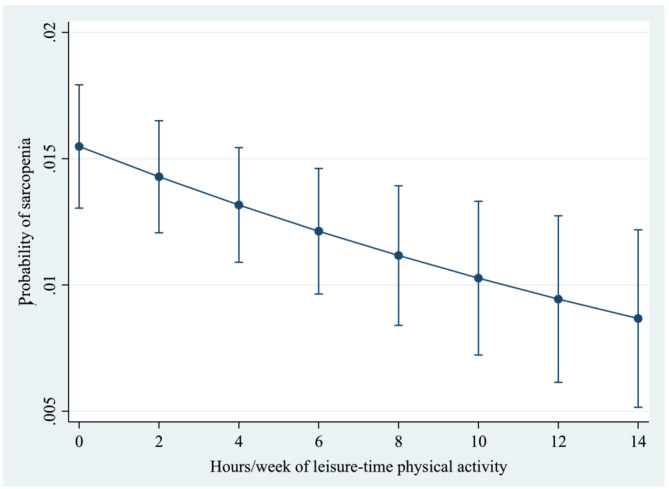
Predicted probability of sarcopenia by h/week of leisure‐time physical activity. Predictions are based on a model adjusted for age, sex, wealth, education, body mass index, number of chronic conditions, activities of daily living difficulty, smoking, alcohol consumption, occupational physical activity, active travel and country, using mean values. Bars denote 95% confidence interval.

The analytical sample consisted of 14 585 individuals aged ≥65 years. The sample size in each country was as follows: China, *n* = 5360; Ghana, *n* = 1975; India, *n* = 2441; Mexico, *n* = 1375; Russia, *n* = 1950; and South Africa, *n* = 1484. Overall, the prevalence of >150 min/week of LTPA and sarcopenia was 8.9% and 12.0%, respectively. The prevalence of >150 min/week of LTPA by country was as follows: China, 14.4%; Ghana, 9.0%; India, 6.3%; Mexico, 2.8%; Russia, 3.9%; and South Africa, 5.2%. The characteristics of the sample are displayed in *Table* *1*. The mean (SD) age was 72.6 (11.5) years, and 55.0% were females. Engagement in ≤150 min/week of LTPA (vs. >150 min/week) was significantly associated with older age, female sex, lower levels of wealth and education, underweight, obesity, ADL difficulty, less alcohol consumption, less occupational physical activity and less active travel. The prevalence of sarcopenia was much higher among those with low levels of LTPA (*Figure* *1*). For example, in the overall sample, the prevalence of sarcopenia among those with >150 and ≤150 min/week of LTPA was 5.9% and 12.6%, respectively. Unadjusted analysis showed that ≤150 min/week of LTPA (vs. >150 min/week) was associated with 2.29 (95% CI = 1.67–3.14), 1.80 (95% CI = 1.23–2.63) and 3.93 (95% CI = 2.33–6.63) times higher odds for sarcopenia in the overall sample, males, and females, respectively (data only shown in the text). After adjustment for potential confounders, ≤150 min/week of LTPA (vs. >150 min/week) was associated with 1.85 (95% CI = 1.29–2.65) times higher odds for sarcopenia, and this was particularly pronounced among women (POR = 3.22; 95% CI = 1.82–5.68) (*Table* *2*). Other types of physical activity (i.e., occupational physical activity and active travel) were not significantly associated with sarcopenia. A 1‐h increase in LTPA per week was associated with 5% lower odds for sarcopenia (POR = 0.95; 95% CI = 0.92–0.98), with relatively similar figures for males (POR = 0.96; 95% CI = 0.93–0.99) and females (POR = 0.92; 95% CI = 0.86–0.98) (data only shown in the text). A visual display of the adjusted predicted probability of sarcopenia by h/week of LTPA based on the overall sample can be found in *Figure* *2*. The sensitivity analysis using a different criterion for weak handgrip strength (i.e., lowest tertile based on country‐ and sex‐stratified values) to define sarcopenia showed that the results were almost the same as the main analysis (*Table* *S2*). The associations between LTPA and the two components of sarcopenia are shown in *Table* *S3* (low SMM) and *Table* *S4* (weak handgrip strength). Significant associations were observed for both measures, but this was sometimes sex specific or exposure specific (i.e., dichotomous or continuous LTPA variable).

**Table 1:** Sample characteristics (overall and by min/week of leisure‐time physical activity)

Characteristic		Leisure‐time physical activity			*P* value^a^
		Overall	>150 min/week	≤150 min/week	
Age (years)	Mean (SD)	72.6 (11.5)	71.0 (9.1)	72.6 (11.5)	<0.001
Sex	Female	55.0	44.6	56.0	<0.001
	Male	45.0	55.4	44.0	
Wealth	Poorest	21.7	14.6	22.3	<0.001
	Poorer	21.0	13.3	21.7	
	Middle	20.4	22.0	20.2	
	Richer	17.5	21.3	17.2	
	Richest	19.4	28.7	18.6	
Education	≤Primary	63.7	49.7	64.8	<0.001
	Secondary	29.9	36.9	29.4	
	Tertiary	6.4	13.4	5.8	
Body mass index (kg/m^2^)	<18.5	19.3	11.4	20.1	<0.001
	18.5–24.9	46.4	52.6	45.7	
	25.0–29.9	23.9	29.5	23.4	
	≥30.0	10.4	6.5	10.8	
Number of chronic conditions	0	16.0	17.8	15.8	0.103
	1	28.7	31.9	28.4	
	≥2	55.3	50.3	55.8	
ADL difficulty	No	88.1	94.5	87.5	0.001
	Yes	11.9	5.5	12.5	
Smoking	Never	62.2	63.3	62.1	0.596
	Past	8.5	9.3	8.4	
	Current	29.3	27.3	29.5	
Alcohol consumption	No	86.1	81.5	86.6	0.003
	Yes	13.9	18.5	13.4	
Occupational physical activity (min/week)	>150	45.8	52.1	45.2	0.025
	≤150	54.2	47.9	54.8	
Active travel (min/week)	>150	37.6	57.0	35.7	<0.001
	≤150	62.4	43.0	64.3	

*Note*: Data are % unless otherwise stated.

Abbreviations: ADL, activities of daily living; SD, standard deviation.

^a^ *P* value was based on chi‐squared tests except for age (Student's *t* test).

**Table 2:** Association between leisure‐time physical activity (and covariates) and sarcopenia (outcome) estimated by multivariable logistic regression (overall and by sex)

Characteristic		Overall		Male		Female	
		POR	95% CI	POR	95% CI	POR	95% CI
Leisure‐time physical activity (min/week)	>150	1.00		1.00		1.00	
	≤150	1.85***	[1.29–2.65]	1.52	[0.99–2.35]	3.22***	[1.82–5.68]
Age (years)	Per 1‐year increase	1.13***	[1.11–1.15]	1.11***	[1.09–1.14]	1.15***	[1.12–1.19]
Sex	Female	1.00					
	Male	1.53***	[1.20–1.95]				
Wealth	Poorest	1.00		1.00		1.00	
	Poorer	0.74	[0.54–1.02]	0.74	[0.47–1.17]	0.80	[0.49–1.31]
	Middle	0.62*	[0.43–0.89]	0.55*	[0.34–0.89]	0.78	[0.49–1.24]
	Richer	0.50***	[0.37–0.67]	0.50**	[0.30–0.83]	0.49***	[0.34–0.72]
	Richest	0.36***	[0.26–0.51]	0.32***	[0.19–0.52]	0.39***	[0.23–0.67]
Education	≤Primary	1.00		1.00		1.00	
	Secondary	0.75	[0.55–1.01]	0.62**	[0.44–0.88]	0.68	[0.33–1.39]
	Tertiary	0.63	[0.36–1.09]	0.57	[0.33–1.01]	0.35	[0.08–1.60]
Body mass index (kg/m^2^)	<18.5	0.59**	[0.40–0.87]	0.31***	[0.18–0.54]	1.17	[0.71–1.91]
	18.5–24.9	1.00		1.00		1.00	
	25.0–29.9	1.46**	[1.11–1.93]	2.53***	[1.80–3.55]	0.92	[0.59–1.41]
	≥30.0	2.04**	[1.33–3.15]	6.21***	[3.12–12.38]	1.00	[0.58–1.72]
Number of chronic conditions	0	1.00		1.00		1.00	
	1	1.23	[0.88–1.72]	1.22	[0.78–1.91]	1.20	[0.67–2.15]
	≥2	1.63**	[1.15–2.33]	1.54	[0.93–2.55]	1.71	[0.95–3.05]
ADL difficulty	No	1.00		1.00		1.00	
	Yes	1.71*	[1.09–2.67]	1.37	[0.82–2.27]	2.08*	[1.07–4.07]
Smoking	Never	1.00		1.00		1.00	
	Past	1.02	[0.70–1.50]	1.02	[0.66–1.58]	0.83	[0.37–1.86]
	Current	0.90	[0.65–1.24]	0.74	[0.52–1.04]	1.39	[0.84–2.28]
Alcohol consumption	No	1.00		1.00		1.00	
	Yes	0.72	[0.48–1.07]	0.69	[0.42–1.12]	0.89	[0.52–1.53]
Occupational physical activity (min/week)	>150	1.00		1.00		1.00	
	≤150	0.84	[0.64–1.10]	0.87	[0.60–1.26]	0.78	[0.55–1.10]
Active travel (min/week)	>150	1.00		1.00		1.00	
	≤150	1.02	[0.79–1.33]	0.97	[0.69–1.36]	1.11	[0.72–1.72]

*Note*: Models are adjusted for all variables in the respective column and country. The *n* for leisure‐time physical activity > 150 and ≤150 min/week was 1204 and 12 994, respectively.

Abbreviations: ADL, activities of daily living; CI, confidence interval; POR, prevalence odds ratio.

* *P* value < 0.05.

** *P* value < 0.01.

*** *P* value < 0.001.

## Discussion

### Main findings

In this cross‐sectional study including more than 14 500 older adults from six LMICs, after adjustment for potential confounders, compared with >150 min/week of LTPA, ≤150 min/week of LTPA was associated with 1.85‐fold (95% CI = 1.29–2.65) higher odds for sarcopenia in the overall sample. Interestingly, the LTPA–sarcopenia relationship was much more pronounced among females, whereas there was no significant association between occupational physical activity or active travel and sarcopenia. To the best of the authors' knowledge, this is the largest study on LTPA and sarcopenia to date, and it is the first study based on data collected in LMICs.

### Interpretation of findings

The finding that LTPA was associated with lower odds for sarcopenia is in line with the only two studies on this topic, which were conducted in high‐income countries.^15^, ^16^ First, in a sample of 122 nursing home older residents in Italy, at least 1 h of LTPA per day was cross‐sectionally associated with lower odds of sarcopenia compared with <1 h of LTPA per day (OR = 0.40; 95% CI = 0.12–0.98).^15^ A second cross‐sectional study, including 1539 older adults living in Spain, found a significant relationship between moderate‐to‐vigorous LTPA and sarcopenia (per 1‐h increase in physical activity per day: prevalence ratio = 0.74; 95% CI = 0.62–0.89).^16^ The present study corroborates these results and adds to the literature by showing that the negative association between LTPA and sarcopenia also exists in LMICs. Compared with the two prior studies, which had relatively small sample sizes and were single‐country studies, our study had a large sample size, was nationally representative and included data collected in six countries, increasing the generalizability of the findings.

There are several hypotheses to explain the relationship between physical activity and sarcopenia. First, physical activity has a direct and positive impact on muscle mass and strength.^13^ A systematic review and meta‐analysis of four randomized controlled trials and three non‐randomized interventional studies (*n* = 235 patients) revealed that exercise had a large effect on physical performance and a medium effect on muscle strength in older adults with sarcopenia.^33^ Second, although the logistic regression analysis was adjusted for chronic conditions, only 11 conditions were considered, and it is possible that other diseases (e.g., cancer) play a mediating role in the association between physical activity and sarcopenia. For example, physical activity is a protective factor against cancer, potentially via effects on hormones and the immune system.^34^ Meanwhile, sarcopenia is a frequent comorbidity in patients with cancer, and cancer can lead to sarcopenia through inflammation, insufficient food intake and dysphagia for certain types of tumours (e.g., head and neck, oesophageal and gastric cancer).^35^ Third, some data suggest that physical activity may reduce risk for polypharmacy via its positive effect on health,^10^ whereas polypharmacy may lead to sarcopenia via drug‐related muscle toxicity, hormonal disturbances and poor nutritional status.^9^ Finally, physically active older individuals are less likely to be hospitalized than those who are not physically active, and this association likely involves fewer chronic conditions, decreased ADL disability and better self‐rated health.^11^ In turn, hospitalization within the past year has been identified as a risk factor for sarcopenia.^12^

It should also be noted that occupational physical activity and active travel were not significantly associated with sarcopenia. LTPA has been found to have stronger positive effects on cardiovascular risk factors (e.g., BMI, body fat and waist circumference) compared with occupational physical activity,^36^ and these stronger effects may explain, at least partially, the different associations by type of physical activity observed in our study. In terms of active travel (e.g., walking or cycling), it is possible that the intensity is not high enough, or it may not be the most ideal form of physical activity to increase muscle mass and strength to prevent sarcopenia in older adults. For example, walking and cycling at a moderate pace may have little effect on upper body muscles compared with leisure activities such as total body resistance training and swimming. Finally, in terms of LTPA, it may be possible to tailor the content of physical activity based on individual needs and focus more on increasing muscle mass and strength than in other types of physical activity, and these specific exercises may be particularly effective in preventing sarcopenia. However, clearly, more research is necessary on the different types of physical activity and sarcopenia, and the underlying mechanisms.

Another important finding of the present study is that the association between low LTPA and sarcopenia was more pronounced among women. Although this sex difference is difficult to explain, some literature indicates that sex differences exist in the epidemiology of sarcopenia,^37^ whereas there are differences in physical activity between women and men.^38^ Furthermore, it is possible for the type and intensity of LTPA to differ between females and males, and females in our study could have been engaging more in LTPA, which is more beneficial in the prevention of sarcopenia. Next, women may be less likely to engage in occupational physical activity and active travel than men, and the effects of LTPA on sarcopenia may therefore be stronger, as their level of physical activity overall may largely be determined by levels of LTPA. Another hypothesis is that, for the same level of LTPA, women may be more health conscientious and consume healthier food than men, which may lead to reduced risk for sarcopenia (e.g., high‐protein diet). However, these explanations are largely speculative, and thus, future research mainly of a qualitative nature is needed to understand these sex differences.

### Public health interventions and directions for future research

Based on the results of this study, LTPA was negatively and significantly associated with sarcopenia in older adults living in LMICs. Several interventions have been recently developed to increase physical activity in older adults.^39^ Some of these interventions can be implemented in LMICs, and these interventions include balance strength/resistance training, physical recreation and health promotion. The prevalence of LTPA was low in the present study, and specific measures should also be taken to encourage regular LTPA in LMICs. These measures should consider sociocultural factors and may involve, for instance, the construction of exercise machines in parks or the promotion of home‐based exercise routines. In terms of future research, more studies of longitudinal nature are warranted to investigate the association between physical activity (including type) and incident sarcopenia in LMICs, while additional data are warranted to better understand potential sex differences in the physical activity–sarcopenia relationship.

### Strengths and limitations of the study

The major strengths of this study are the large sample size, the use of nationally representative data collected in six LMICs and the analysis of different types of physical activity (i.e., LTPA, occupational physical activity and active travel). Nonetheless, the study findings should be interpreted in light of several limitations. First, data on physical activity were self‐reported, potentially biasing some of the findings of the study. Second, SMM was estimated based on an equation. That being said, there is good concordance between the equation and direct methods, such as dual‐energy X‐ray absorptiometry and magnetic resonance imaging.^22^ Third, there was no information on nutritional status, despite the fact that this is an important determinant of sarcopenia. Thus, some level of residual confounding due to this factor may exist. Fourth, we were unable to conduct country‐wise analyses as meaningful estimates could not be obtained due to small sample size in each country and lack of statistical power. Future studies with larger sample size should consider conducting country‐wise analyses to assess whether associations are context specific. Fifth, there is no established cut‐off to define low LTPA. Thus, the cut‐off used in our main analysis (150 min/week) should not necessarily be interpreted as the optimal cut‐off for sarcopenia prevention. Sixth, due to a lack of data, we were unable to conduct analyses on the association between sarcopenia and different categories of sports, fitness and recreational activities. Finally, because this was a cross‐sectional study, it was not possible to investigate the temporal association between LTPA and sarcopenia.

## Conclusions

LTPA was negatively and significantly associated with sarcopenia among older adults in LMICs, with this association potentially being more pronounced among women. Promotion of LTPA may be important in the prevention of sarcopenia among older adults in LMICs, but future longitudinal and interventional studies are necessary before concrete recommendations can be made.

## Conflict of interest

The authors declare no conflicts of interest.

## Supporting information


**Table S1.** Details on the diagnosis of the 11 chronic conditions
**Table S2.** Sensitivity analysis on the association between leisure‐time physical activity and sarcopenia (outcome) estimated by multivariable logistic regression using a different criterion of weak handgrip strength (overall and by sex)
**Table S3.** Association between leisure‐time physical activity and low skeletal muscle mass (outcome) estimated by multivariable logistic regression (overall and by sex)
**Table S4.** Association between leisure‐time physical activity and weak handgrip strength (outcome) estimated by multivariable logistic regression (overall and by sex)

## References

[1] Landi F , Calvani R , Cesari M , Tosato M , Martone AM , Ortolani E , et al. Sarcopenia: an overview on current definitions, diagnosis and treatment. Curr Protein Pept Sci 2018;19:633–638. 28595526 10.2174/1389203718666170607113459

[2] Daskalopoulou C , Wu Y‐T , Pan W , Giné Vázquez I , Prince M , Prina M , et al. Factors related with sarcopenia and sarcopenic obesity among low‐ and middle‐income settings: the 10/66 DRG study. Sci Rep 2020;10:20453. 33235211 10.1038/s41598-020-76575-4 PMC7686337

[3] World Health Organization . Ageing. https://www.who.int/health‐topics/ageing#tab=tab_1 (2022). Accessed 2 October 2022.

[4] Chien M‐Y , Kuo H‐K , Wu Y‐T . Sarcopenia, cardiopulmonary fitness, and physical disability in community‐dwelling elderly people. Phys Ther 2010;90:1277–1287. 20616117 10.2522/ptj.20090322

[5] Gao K , Ma W‐Z , Huck S , Li B‐L , Zhang L , Zhu J , et al. Association between sarcopenia and depressive symptoms in Chinese older adults: evidence from the China Health and Retirement Longitudinal Study. Front Med (Lausanne) 2021;8:755705. 34869454 10.3389/fmed.2021.755705 PMC8635632

[6] Bunout D , de la Maza MP , Barrera G , Leiva L , Hirsch S . Association between sarcopenia and mortality in healthy older people. Australas J Ageing 2011;30:89–92. 21672118 10.1111/j.1741-6612.2010.00448.x

[7] Dasso NA . How is exercise different from physical activity? A concept analysis. Nurs Forum 2019;54:45–52. 30332516 10.1111/nuf.12296

[8] Pacifico J , Geerlings MAJ , Reijnierse EM , Phassouliotis C , Lim WK , Maier AB . Prevalence of sarcopenia as a comorbid disease: a systematic review and meta‐analysis. Exp Gerontol 2020;131:110801. 31887347 10.1016/j.exger.2019.110801

[9] König M , Spira D , Demuth I , Steinhagen‐Thiessen E , Norman K . Polypharmacy as a risk factor for clinically relevant sarcopenia: results from the Berlin Aging Study II. J Gerontol A Biol Sci Med Sci 2017;73:117–122. 28481965 10.1093/gerona/glx074

[10] Thanoo N , Gilbert AL , Trainor S , Semanik PA , Song J , Lee J , et al. The relationship between polypharmacy and physical activity in those with or at risk of knee osteoarthritis. J Am Geriatr Soc 2020;68:2015–2020. 32441333 10.1111/jgs.16501 PMC7680293

[11] Li C‐L , Chu S‐J , Sheu J‐T , Huang LY‐G . Impact of physical activity on hospitalization in older adults: a nationwide cohort from Taiwan. Arch Gerontol Geriatr 2011;53:141–145. 21067830 10.1016/j.archger.2010.09.014

[12] Kitamura A , Seino S , Abe T , Nofuji Y , Yokoyama Y , Amano H , et al. Sarcopenia: prevalence, associated factors, and the risk of mortality and disability in Japanese older adults. J Cachexia Sarcopenia Muscle 2021;12:30–38. 33241660 10.1002/jcsm.12651 PMC7890144

[13] Steffl M , Bohannon RW , Sontakova L , Tufano JJ , Shiells K , Holmerova I . Relationship between sarcopenia and physical activity in older people: a systematic review and meta‐analysis. Clin Interv Aging 2017;12:835–845. 28553092 10.2147/CIA.S132940 PMC5441519

[14] Liu W . Editorial: barriers to promoting physical activity in low‐ and middle‐income countries: alignment with the sustainable development goals. Front Public Health 2022;10:943428. 35757647 10.3389/fpubh.2022.943428 PMC9221515

[15] Landi F , Liperoti R , Fusco D , Mastropaolo S , Quattrociocchi D , Proia A , et al. Prevalence and risk factors of sarcopenia among nursing home older residents. J Gerontol A Biol Sci Med Sci 2012;67:48–55. 21393423 10.1093/gerona/glr035

[16] Rosique‐Esteban N , Babio N , Díaz‐López A , Romaguera D , Alfredo Martínez J , Sanchez VM , et al. Leisure‐time physical activity at moderate and high intensity is associated with parameters of body composition, muscle strength and sarcopenia in aged adults with obesity and metabolic syndrome from the PREDIMED‐Plus study. Clin Nutr 2019;38:1324–1331. 29910068 10.1016/j.clnu.2018.05.023

[17] da Silva ICM , Mielke GI , Bertoldi AD , Arrais PSD , Luiza VL , Mengue SS , et al. Overall and leisure‐time physical activity among Brazilian adults: national survey based on the Global Physical Activity Questionnaire. J Phys Act Health 2018;15:212–218. 28872402 10.1123/jpah.2017-0262

[18] Kowal P , Chatterji S , Naidoo N , Biritwum R , Fan W , Lopez Ridaura R , et al. Data resource profile: the World Health Organization Study on global AGEing and adult health (SAGE). Int J Epidemiol 2012;41:1639–1649. 23283715 10.1093/ije/dys210 PMC3535754

[19] Cruz‐Jentoft AJ , Bahat G , Bauer J , Boirie Y , Bruyère O , Cederholm T , et al. Sarcopenia: revised European consensus on definition and diagnosis. Age Ageing 2019;48:16–31. 30312372 10.1093/ageing/afy169 PMC6322506

[20] Lee RC , Wang Z , Heo M , Ross R , Janssen I , Heymsfield SB . Total‐body skeletal muscle mass: development and cross‐validation of anthropometric prediction models. Am J Clin Nutr 2000;72:796–803. 10966902 10.1093/ajcn/72.3.796

[21] Studenski SA , Peters KW , Alley DE , Cawthon PM , McLean RR , Harris TB , et al. The FNIH sarcopenia project: rationale, study description, conference recommendations, and final estimates. J Gerontol A Biol Sci Med Sci 2014;69:547–558. 24737557 10.1093/gerona/glu010 PMC3991146

[22] Tyrovolas S , Koyanagi A , Olaya B , Ayuso‐Mateos JL , Miret M , Chatterji S , et al. Factors associated with skeletal muscle mass, sarcopenia, and sarcopenic obesity in older adults: a multi‐continent study. J Cachexia Sarcopenia Muscle 2016;7:312–321. 27239412 10.1002/jcsm.12076 PMC4864288

[23] Ortiz O , Russell M , Daley TL , Baumgartner RN , Waki M , Lichtman S , et al. Differences in skeletal muscle and bone mineral mass between black and white females and their relevance to estimates of body composition. Am J Clin Nutr 1992;55:8–13. 1728823 10.1093/ajcn/55.1.8

[24] Bull FC , Maslin TS , Armstrong T . Global Physical Activity Questionnaire (GPAQ): nine country reliability and validity study. J Phys Act Health 2009;6:790–804. 20101923 10.1123/jpah.6.6.790

[25] Laverty AA , Palladino R , Lee JT , Millett C . Associations between active travel and weight, blood pressure and diabetes in six middle income countries: a cross‐sectional study in older adults. Int J Behav Nutr Phys Act 2015;12:65. 25986001 10.1186/s12966-015-0223-3 PMC4443597

[26] Katz S , Ford AB , Moskowitz RW , Jackson BA , Jaffe MW . Studies of illness in the aged: the index of ADL: a standardized measure of biological and psychosocial function. JAMA 1963;185:914–919. 14044222 10.1001/jama.1963.03060120024016

[27] Al Snih S , Graham JE , Kuo Y‐F , Goodwin JS , Markides KS , Ottenbacher KJ . Obesity and disability: relation among older adults living in Latin America and the Caribbean. Am J Epidemiol 2010;171:1282–1288. 20472569 10.1093/aje/kwq087 PMC2915495

[28] Backholer K , Wong E , Freak‐Poli R , Walls HL , Peeters A . Increasing body weight and risk of limitations in activities of daily living: a systematic review and meta‐analysis. Obes Rev 2012;13:456–468. 22212629 10.1111/j.1467-789X.2011.00970.x

[29] Koyanagi A , Moneta MV , Garin N , Olaya B , Ayuso‐Mateos JL , Chatterji S , et al. The association between obesity and severe disability among adults aged 50 or over in nine high‐income, middle‐income and low‐income countries: a cross‐sectional study. BMJ Open 2015;5:e007313. 10.1136/bmjopen-2014-007313 PMC4390733 25838510

[30] Smith L , Veronese N , López‐Sánchez GF , Yang L , Pizzol D , Butler LT , et al. Active travel and mild cognitive impairment among older adults from low‐ and middle‐income countries. J Clin Med 2021;10:1243. 33802825 10.3390/jcm10061243 PMC8002501

[31] Koyanagi A , Garin N , Olaya B , Ayuso‐Mateos JL , Chatterji S , Leonardi M , et al. Chronic conditions and sleep problems among adults aged 50 years or over in nine countries: a multi‐country study. PLoS ONE 2014;9:e114742. 25478876 10.1371/journal.pone.0114742 PMC4257709

[32] Koyanagi A , Lara E , Stubbs B , Carvalho AF , Oh H , Stickley A , et al. Chronic physical conditions, multimorbidity, and mild cognitive impairment in low‐ and middle‐income countries. J Am Geriatr Soc 2018;66:721–727. 29427504 10.1111/jgs.15288 PMC5906176

[33] Escriche‐Escuder A , Fuentes‐Abolafio IJ , Roldán‐Jiménez C , Cuesta‐Vargas AI . Effects of exercise on muscle mass, strength, and physical performance in older adults with sarcopenia: a systematic review and meta‐analysis according to the EWGSOP criteria. Exp Gerontol 2021;151:111420. 34029642 10.1016/j.exger.2021.111420

[34] de Rezende LFM , de Sá TH , Markozannes G , Rey‐López JP , Lee I‐M , Tsilidis KK , et al. Physical activity and cancer: an umbrella review of the literature including 22 major anatomical sites and 770 000 cancer cases. Br J Sports Med 2018;52:826–833. 29146752 10.1136/bjsports-2017-098391

[35] Surov A , Wienke A . Prevalence of sarcopenia in patients with solid tumors: a meta‐analysis based on 81,814 patients. JPEN J Parenter Enteral Nutr 2022;46:1761–1768. 35633306 10.1002/jpen.2415

[36] Oppert J‐M , Thomas F , Charles M‐A , Benetos A , Basdevant A , Simon C . Leisure‐time and occupational physical activity in relation to cardiovascular risk factors and eating habits in French adults. Public Health Nutr 2006;9:746–754. 16925880 10.1079/phn2005882

[37] Tay L , Ding YY , Leung BP , Ismail NH , Yeo A , Yew S , et al. Sex‐specific differences in risk factors for sarcopenia amongst community‐dwelling older adults. Age (Dordr) 2015;37:121. 26607157 10.1007/s11357-015-9860-3 PMC5005859

[38] Li W , Procter‐Gray E , Churchill L , Crouter SE , Kane K , Tian J , et al. Gender and age differences in levels, types and locations of physical activity among older adults living in car‐dependent neighborhoods. J Frailty Aging 2017;6:129–135. 28721428 10.14283/jfa.2017.15 PMC5612373

[39] Taylor J , Walsh S , Kwok W , Pinheiro MB , de Oliveira JS , Hassett L , et al. A scoping review of physical activity interventions for older adults. Int J Behav Nutr Phys Act 2021;18:82. 34193157 10.1186/s12966-021-01140-9 PMC8243293

[40] von Haehling S , Morley JE , Coats AJS , Anker SD . Ethical guidelines for publishing in the Journal of Cachexia, Sarcopenia and Muscle: update 2021. J Cachexia Sarcopenia Muscle 2021;12:2259–2261. 34904399 10.1002/jcsm.12899 PMC8718061

