# Supplementary material for: Leisure‐time physical activity and sarcopenia among older adults from low‐ and middle‐income countries
Source: J Cachexia Sarcopenia Muscle. 2023 Mar 5;14(2):1130–8. doi: 10.1002/jcsm.13215 (PMC10067478; doi:10.1002/jcsm.13215)
Supplement: Supplementary file 1 — Table S1. Details on the diagnosis of the 11 chronic conditions Table S2. Sensitivity analysis on the association between leisure‐time physical activity and sarcopenia (outcome) estimated by multivariable logistic regression using a different criterion of weak handgrip strength (overall and by sex) Table S3. Association between leisure‐time physical activity and low skeletal muscle mass (outcome) estimated by multivariable logistic regression (overall and by sex) Table S4. Association between leisure‐time physical activity and weak handgrip strength (outcome) estimated by multivariable logistic regression (overall and by sex) [file JCSM-14-1130-s001.docx]

**Appendix**

**Table S1.** Details on the diagnosis of the 11 chronic conditions

| Condition | (a) Self-reported diagnosis or symptoms | (b) Symptom-based algorithm or other method of diagnosis |
| --- | --- | --- |
| Angina | Have you ever been diagnosed with angina or angina pectoris (a heart disease)? | Rose questionnaire |
| Arthritis | Have you ever been diagnosed with/told you have arthritis (a disease of the joints, or by other names rheumatism or osteoarthritis)? | Not applicable |
| Asthma | Have you ever been diagnosed with asthma (an allergic respiratory disease)? | Not applicable |
| Chronic back pain | Back pain everyday during the last 30 days. | Not applicable |
| Chronic lung disease | Have you ever been diagnosed with chronic lung disease (emphysema, bronchitis, COPD)? | Not applicable |
| Diabetes | Have you ever been diagnosed with diabetes (high blood sugar)? (not including diabetes associated with a pregnancy) | Not applicable |
| Edentulism | Have you lost all of your natural teeth? | Not applicable |
| Hearing problem | Not applicable | Interviewer observation |
| Hypertension | Have you ever been diagnosed with high blood pressure (hypertension)? | Blood pressure was measured three times with a one-minute interval with the use of a wrist blood pressure monitor (Medistar Wrist Blood Pressure Model S), and the mean value of the three measurements was calculated. Hypertension was defined as having at least one of the following: systolic blood pressure ≥140 mmHg; diastolic blood pressure ≥90 mmHg. |
| Stroke | Have you ever been told by a health professional that you have had a stroke? | Not applicable |
| Visual impairment | Extreme difficulty in seeing and recognizing a person that the participant knows across the road | Not applicable |

Abbreviation: COPD chronic obstructive pulmonary disease.

**Table S2.** Sensitivity analysis on the association between leisure-time physical activity and sarcopenia (outcome) estimated by multivariable logistic regression using a different criterion of weak handgrip strength (overall and by sex)

|  |  | Overall | | Male | | Female | |
| --- | --- | --- | --- | --- | --- | --- | --- |
|  |  | POR | 95%CI | POR | 95%CI | POR | 95%CI |
| ≤150 minutes/week of LTPA | No | 1.00 |  | 1.00 |  | 1.00 |  |
|  | Yes | 1.99*** | [1.32,2.99] | 1.47 | [0.91,2.35] | 3.65*** | [1.87,7.12] |
| LTPA (per hour/week) | Per one-hour increase | 0.95** | [0.92,0.98] | 0.96* | [0.93,1.00] | 0.92* | [0.86,0.98] |

Abbreviations: POR prevalence odds ratio; CI confidence interval; LTPA leisure-time physical activity.

Models are adjusted for age, sex, wealth, education, body mass index, number of chronic conditions, ADL difficulty, smoking, alcohol consumption, occupational physical activity, active travel, and country, with the exception of the sex-stratified analysis which was not adjusted for sex.

Low handgrip strength was defined as the lowest tertile of handgrip strength based on sex- and country-stratified values.

* p<0.05, ** p<0.01, *** p<0.001.

**Table S3.** Association between leisure-time physical activity and low skeletal muscle mass (outcome) estimated by multivariable logistic regression (overall and by sex)

|  |  | Overall | | Male | | Female | |
| --- | --- | --- | --- | --- | --- | --- | --- |
|  |  | POR | 95%CI | POR | 95%CI | POR | 95%CI |
| ≤150 minutes/week of LTPA | No | 1.00 |  | 1.00 |  | 1.00 |  |
|  | Yes | 1.33 | [0.94,1.88] | 1.29 | [0.84,1.96] | 1.62* | [1.00,2.63] |
| LTPA (per hour/week) | Per one-hour increase | 0.96** | [0.93,0.99] | 0.96* | [0.93,0.99] | 0.95* | [0.91,1.00] |

Abbreviations: POR prevalence odds ratio; CI confidence interval; LTPA leisure-time physical activity.

Models are adjusted for age, sex, wealth, education, body mass index, number of chronic conditions, ADL difficulty, smoking, alcohol consumption, occupational physical activity, active travel, and country, with the exception of the sex-stratified analysis which was not adjusted for sex.

* p<0.05, ** p<0.01.

**Table S4.** Association between leisure-time physical activity and weak handgrip strength (outcome) estimated by multivariable logistic regression (overall and by sex)

|  |  | Overall | | Male | | Female | |
| --- | --- | --- | --- | --- | --- | --- | --- |
|  |  | POR | 95%CI | POR | 95%CI | POR | 95%CI |
| ≤150 minutes/week of LTPA | No | 1.00 |  | 1.00 |  | 1.00 |  |
|  | Yes | 1.59** | [1.20,2.10] | 1.37 | [0.97,1.94] | 1.91** | [1.19,3.07] |
| LTPA (per hour/week) | Per one-hour increase | 0.98 | [0.94,1.03] | 0.97* | [0.95,1.00] | 1.00 | [0.92,1.07] |

Abbreviations: POR prevalence odds ratio; CI confidence interval; LTPA leisure-time physical activity.

Models are adjusted for age, sex, wealth, education, body mass index, number of chronic conditions, ADL difficulty, smoking, alcohol consumption, occupational physical activity, active travel, and country, with the exception of the sex-stratified analysis which was not adjusted for sex.

* p<0.05, ** p<0.01.
